# Re-description of *Clubionahuaban* (Araneae, Clubionidae), with the first description of the female

**DOI:** 10.3897/BDJ.13.e157384

**Published:** 2025-07-11

**Authors:** Jianshuang Zhang, Qiwen Du, Yingchen Dai, Hao Yu

**Affiliations:** 1 School of Life Sciences, Guizhou Normal University, Guiyang, China School of Life Sciences, Guizhou Normal University Guiyang China

**Keywords:** DNA barcoding, sac spiders, morphology, taxonomy

## Abstract

**Background:**

*Clubionahuaban* Xin, Zhang, Li, Zeng & Yu, 2020, one of the members of the *Clubionatrivialis* group, was described, based on two male types from Xiangzhigou Scenic Spot, Guyang City, Guizhou Province, China.

**New information:**

Recently, new material of sac spiders has been collected from southwest China, including the type locality. The males were identified as *C.huaban*, based on comparison with the holotype. On the basis of the morphological characters and DNA barcoding, we credibly matched the females and males together as *C.huaban*. Therefore, *C.huaban* is re-described, based on new material and the female is described and illustrated for the first time.

## Introduction

The genus *Clubiona* Latreille, 1804, the type genus of the family Clubionidae, currently comprises 531 extant species distributed across most tropical and temperate regions worldwide, accounting for approximately 79% of the total species within the family (WSC 2025). Owing to the high species diversity within *Clubiona*, numerous efforts have been made to divide the genus into distinct genera, subgenera or species groups (Marusik and Omelko 2018; Zhang et al. 2021).

The *Clubionatrivialis* group was initially proposed by Edwards (1958) and formally established by Dondale and Redner (1976). Mikhailov (1995) further refined the group, recognising 19 *Clubiona* species from the Holarctic Region. The diagnosis of the *C.trivialis* group has been repeatedly addressed by various taxonomists and the composition of its core members has remained relatively stable over time (Dondale and Redner 1976, Mikhailov 1995, Yu and Li 2019a, Yu and Li 2019b, Zhang et al. 2021). Species of the *Clubionatrivialis* group can be identified by a distinctive combination of morphological features (see also Dondale and Redner (1976), Mikhailov (1995), Yu and Li (2019a)). In males, retrolateral tibial apophysis simple, erect and lacks prominent dentition; embolic base typically bears one to three tooth-like processes; embolus arched around or angled across the distal end of tegulum; conductor membranous, groove-like, distally situated on the retrolateral side of tegulum and fused to it; sperm duct clearly visible and sinuous. In females, epigyne comprises large ventral plate and semi-transparent dorsal plate; ventral plate disc-shaped, with strongly sclerotised posterior margin extending beyond epigastric furrow; copulatory openings positioned posteriorly on ventral plate, joined medially or spaced closely; copulatory ducts slender, extending laterally in straight or arched paths and run parallel and close together at mid-line; both primary spermathecae and secondary spermathecae sandwiched between the dorsal and ventral plates.

The latest species list of the *trivialis*-group was provided by Zhang et al. (2021), including 28 species. After that, two additional new species were described from China by Li et al. (2023) and Zhang et al. (2024): *C.flammaformis* L. F. Li, Liu, B. Li & Peng, 2023 and *C.bi* Zhang, Zhong & Gong, 2024. In addition, *C.hooda* Dong & Zhang, 2016, which was originally assigned to the *trivialis* group by Dong and Zhang (2016), was transferred to the *zilla* group by Zeng et al. (2021). To date, the *trivialis* group comprises at least 29 species, with a primary distribution across Eurasia and Australia, 17 of which are known to occur in China (Suppl. material 1). Amongst these, six have been described based on a single sex only and COI barcode sequences are available from GenBank for only 11 species (Suppl. material 1).

**Figure 1.**
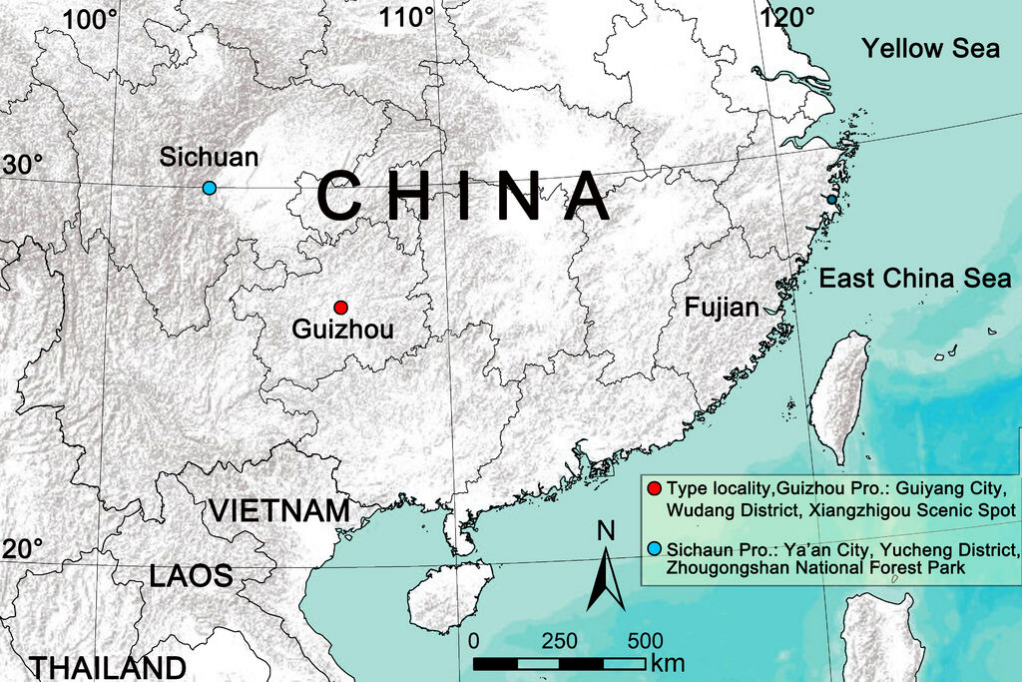
Distribution records of *Clubionahuaban*.

The species *Clubionahuaban* Xin, Zhang, Li, Zeng & Yu, 2020, one of the members of the *Clubionatrivialis* group, was originally described based on two male specimens from Guiyang City, Guizhou Province, China (Xin et al. 2020). Recently, the authors examined *Clubiona* specimens collected from southwest China, including Xiangzhigou Scenic Spot in Guiyang City (the type locality of *C.huaban*) and Ya’an City, Sichuan Province (Fig. 1). Several specimens, representing both sexes, were found at the same locations, exhibiting similar habitus, markings, leg spination and other morphological characteristics. This strongly suggests that these individuals represent the opposite sexes of the same species. Based on comparison with the type specimen, we identified the male as *C.huaban*. DNA barcodes (a partial fragment of the mitochondrial cytochrome oxidase subunit I gene, COI) of the new materials of *C.huaban* was also obtained to confirm gender matching and species identification (Table 1；Suppl. material 1). A preliminary molecular species delimitation was conducted using the DNA barcoding gap method, based on all species for which COI sequences were available (including newly-generated sequences of *C.huaban* and additional sequences of other species obtained from NCBI). The aim of the current paper is to: 1) evaluate the effectiveness of COI sequences as DNA barcodes for species delimitation within the *Clubionatrivialis* group; and (2) re-describe the male and report the female of *C.huaban* for the first time, providing detailed morphological descriptions and illustrations, along with the first COI sequence for the species.

## Materials and methods

**Taxon sampling**

Specimens of *C.huaban* in this study were collected by hand and then the right legs were removed to be stored at −80°C for subsequent DNA extraction. The remainder of the specimens was preserved in 80% ethanol for identification and morphological examination. A total of eight adults were obtained, examined and processed for DNA extraction. However, we were unable to obtain high-quality DNA extractions from four samples. Finally, we sampled four individuals for molecular species delimitation (Table 1; Suppl. material 1).

To conduct the molecular species delimitation analysis using the DNA barcoding gap method, we also retrieved a number of sequences from NCBI, including all 11 species within the *Clubionatrivialis* group for which COI sequences are publicly available (Suppl. material 1). The criteria for sequence selection were as follows: (1) only sequences amplified using the primers LCOI1490 and HCOI2198 were included; (2) for species with more than five available sequences, such as *C.trivialis* and *C.diversa*, five sequences were randomly selected, while, for species with fewer than five sequences, such as *C.subquebecana* and *C.subasrevida*, all available sequences were included. A total of 38 sequences from 11 species were ultimately obtained from NCBI for inclusion in the molecular species delimitation analysis (accession numbers are provided in Suppl. material 1).

**Molecular protocols**

A DNA barcode was also obtained for the species matching and species delimitation. A partial fragment of the mitochondrial cytochrome oxidase subunit I (CO1) gene was amplified and sequenced for one male and one female specimen, respectively, using the primers LCOI1490 (5’-GGTCAACAAATCATAAAGATATTG-3’) and HCOI2198 (5’-TAAACTTCAGGGTGACCAAAAAAT-3’). For additional information on extraction, amplification and sequencing procedures, see Malumbres-Olarte and Vink (2012). Raw sequences were edited and assembled using BioEdit v.7.2.5 (Hall 1999) and uncorrected pairwise distances between sequences were calculated using MEGA v.10.0 (Tamura et al. 2013). All sequences were analysed using BLAST and are deposited in GenBank.

**Molecular species delimitation**

In order to delimit 12 putative morphospecies of the *trivialis*-group, based on an accompanying morphological study of the group, we conducted a genetic distance-based method: the DNA barcoding gap (Barrett and Hebert 2005). We examined the overlap between the interspecies and intraspecies Kimura two-parameter (K2P) and uncorrected p-distance for each candidate species calculated in MEGA X (Kumar et al. 2018).

**Morphological protocols**

Specimens were examined with an Olympus SZX7 stereomicroscope; details were studied with an Olympus BX41 compound microscope. Male palps and female epigynes were examined and illustrated after being dissected. Epigynes were removed and cleared in warm lactic acid before illustration. Photos were made with a Canon EOS70D digital camera mounted on an Olympus CX41 compound microscope. The digital images were taken and assembled using Helifocus 3.10.3. software package (Khmelik et al. 2005).

All measurements were obtained using an Olympus SZX7 stereomicroscope and given in millimetres. Eye diameters were measured at the widest part. The total body length does not include the chelicerae or spinnerets. Leg lengths are given as total length (femur, patella + tibia, metatarsus, tarsus).

The distribution map was generated with ArcGIS ver. 10.5 (Environmental Systems Research Institute, Inc.). The terminology used in the text and figure legends follows Yu and Li (2019a), Yu and Li (2019b), Xin et al. (2020) and Zhang et al. (2021). Abbreviations used in the text and figures are as follows: **AER** anterior eye row; **ALE** anterior lateral eyes; **AME** anterior median eyes; **AME–AME** distance between AMEs; **AME–ALE** distance between AME and ALE; **BS** bursa; **C** conductor; **CD** copulatory ducts; **CO** copulatory opening; **Cy** cymbium; **EB** embolar base; **EBP** embolar base process; **Em** embolus; **ET** embolar tip; **FD** fertilisation duct; **MOQ** median ocular quadrangle; **MOQA** MOQ anterior width; **MOQL** length of MOQ; **MOQP** MOQ posterior width; **PER** posterior eye row; **PLE** posterior lateral eyes; **PME** posterior median eyes; **PME–PME** distance between PMEs; **PME–PLE** distance between PME and PLE; **PA** patellar apophysis; **RTA** retrolateral tibial apophysis; **SD** sperm duct; **SG** spermathecal gland; **Sp1** primary spermatheca; **Sp2** secondary spermatheca; **St** subtegulum; **Te** tegulum; **TH** tegular hump.

## Taxon treatments

### Clubiona huaban

Xin, Zhang, Li, Zeng & Yu, 2020

7ACF8B40-09C5-5C8B-8C1A-A7C770F8FF96

#### Materials

- **Type status:** Holotype. **Occurrence:** recordedBy: Hao Yu; individualCount: 1; sex: 1 male; lifeStage: 1 adult; preparations: whole animal (ETOH); occurrenceID: 038DEA2D-12E1-5F61-A132-FB2D4F38DE97; **Taxon:** scientificName: *Clubionahuaban*; order: Araneae; family: Clubionidae; genus: Clubiona; specificEpithet: *huaban*; taxonRank: species; scientificNameAuthorship: Xin, Zhang, Li, Zeng & Yu, 2020; taxonomicStatus: accepted; **Location:** continent: Asia; country: China; countryCode: CHN; stateProvince: Guizhou; county: Wudang; municipality: Guiyang; locality: Xiangzhigou Scenic Spot; verbatimElevation: 1115 m; decimalLatitude: 26.786153; decimalLongitude: 106.921736; georeferenceProtocol: label; **Identification:** identifiedBy: Hao Yu; dateIdentified: 11-11-2024; identificationReferences: Xin et al. 2020; **Event:** samplingProtocol: beating; samplingEffort: 10 km by foot; eventDate: 20/5/2016; year: 2016; month: 5; day: 20; **Record Level:** language: en; basisOfRecord: PreservedSpecimen
- **Type status:** Other material. **Occurrence:** recordedBy: Hao Yu; individualCount: 3; sex: 1 male, 2 females; lifeStage: 3 adults; preparations: whole animal (ETOH); associatedSequences: PV868258; PV868259; occurrenceID: 270003E3-CB97-5F07-9B2D-FFBDB2BA607C; **Taxon:** scientificName: *Clubionahuaban*; order: Araneae; family: Clubionidae; genus: Clubiona; specificEpithet: *huaban*; taxonRank: species; scientificNameAuthorship: Xin, Zhang, Li, Zeng & Yu, 2020; taxonomicStatus: accepted; **Location:** continent: Asia; country: China; countryCode: CHN; stateProvince: Guizhou; county: Wudang; municipality: Guiyang; locality: Xiangzhigou Scenic Spot; verbatimElevation: 1115 m; decimalLatitude: 26.786153; decimalLongitude: 106.921736; georeferenceProtocol: label; **Identification:** identifiedBy: Hao Yu; dateIdentified: 11-11-2024; identificationReferences: Xin et al. 2020; **Event:** samplingProtocol: beating; samplingEffort: 10 km by foot; eventDate: 20/6/2023; year: 2023; month: 6; day: 20; **Record Level:** language: en; basisOfRecord: PreservedSpecimen
- **Type status:** Other material. **Occurrence:** recordedBy: Hao Yu; individualCount: 4; sex: 2 male, 2 female; lifeStage: 4 adults; preparations: whole animal (ETOH); associatedSequences: PV868257; PV868260; occurrenceID: CCD9B95A-05D5-53B6-B78C-2867514B2491; **Taxon:** scientificName: *Clubionahuaban*; order: Araneae; family: Clubionidae; genus: Clubiona; specificEpithet: *huaban*; taxonRank: species; scientificNameAuthorship: Xin, Zhang, Li, Zeng & Yu, 2020; taxonomicStatus: accepted; **Location:** continent: Asia; country: China; countryCode: CHN; stateProvince: Sichuan; county: Yucheng; municipality: Ya’an; locality: Zhougongshan National Forest Park; verbatimElevation: 650 m; decimalLatitude: 29.959043; decimalLongitude: 103.047465; georeferenceProtocol: label; **Identification:** identifiedBy: Hao Yu; dateIdentified: 11-11-2024; identificationReferences: Xin et al. 2020; **Event:** samplingProtocol: beating; samplingEffort: 10 km by foot; eventDate: 22/5/2023; year: 2023; month: 5; day: 22; **Record Level:** language: en; basisOfRecord: PreservedSpecimen

#### Description

**Figure 2.**
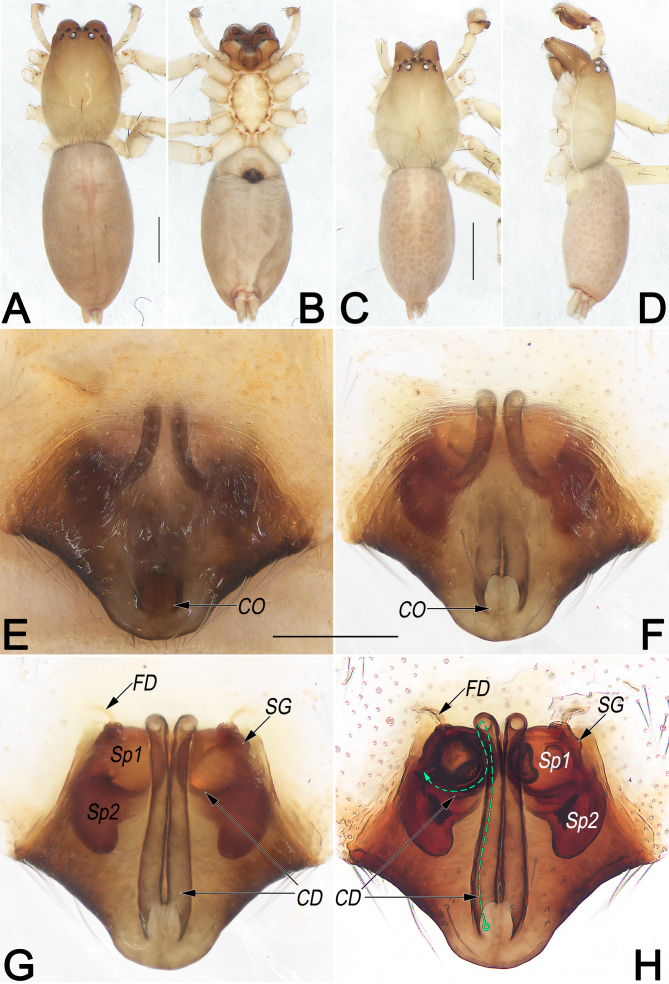
*Clubionahuaban*, female and male from Guiyang, female habitus (**A, B**), male habitus (**C, D**) and epigyne (**E–H**). **A** Dorsal view; **B** Ventral view; **C** Dorsal view; **D** Lateral view; **E** Intact, ventral view; **F** Cleared and macerated, ventral view; **G** Cleared and macerated, dorsal view; **H** Cleared and embedded in Arabic gum, dorsal view (dashed line in H showing schematic course of copulatory duct, dorsal). Abbreviations: CD = copulatory ducts; CO = copulatory opening; FD = fertilisation duct; SG = spermathecal gland; Sp1 = primary spermatheca; Sp2 = secondary spermatheca. Scale bars: 1 mm (equal for **A, B**, equal for **C, D**); 0.2 mm (equal for **E–H**).

**Female (Fig. 2A, B)**. Total length 6.47; carapace 2.59 long, 1.77 wide; abdomen 3.88 long, 2.04 wide. Eye sizes and interdistances: AME 0.13, ALE 0.16, PME 0.12, PLE 0.13, AME–AME 0.08, AME–ALE 0.07, PME–PME 0.29, PME–PLE 0.17, MOQL 0.35, MOQA 0.33, MOQP 0.55. Sternum 1.33 long, 0.84 wide. Leg measurements: I 5.63 (1.54, 2.39, 1.14, 0.56), II 5.93 (1.71, 2.47, 1.15, 0.60), III 4.98 (1.47, 1.73, 1.30, 0.48), IV 7.60 (2.15, 2.66, 2.19, 0.60). Leg formula: IV-II-I-III. Cheliceral furrow with six promarginal and three retromarginal teeth.

**Colouration in ethanol** (Fig. 2A and B). Carapace light brown, without distinct pattern, fovea reddish; cephalic region slightly narrowed and slightly darker, cervical groove and radial grooves indistinct; tegument smooth, marginally clothed with short, fine hairs. Eyes: in dorsal view, AER slightly recurved, PER almost straight, the latter wider than the former. Chelicerae robust and light reddish-brown. Sternum yellowish-white. Labium and endites light orange. Legs coloured as sternum, without distinct markings. Abdomen elongate, oval, with a thick tuft of setae anteriorly; dorsally with a lengthwise pink heart mark which is shaped like double-edged spear, reaching posterior half. Venter uniformly creamy white, without markings.

**Epigyne** (Fig. 2E–H). Epigynal plate ca. 1.3× wider than long, anterior and lateral margin not rebordered; posterior margin heavily sclerotised and convex medially, with a downward-protruding edge, forming an inverted trapezoidal outline; spermathecae and copulatory ducts are prominently through epigynal plate in ventral view. Copulatory openings (CO) close together, apple-shaped, moderately large, ca. 1/6 of epigyne length and 1/9 of epigyne width. Copulatory ducts (CD) long and slender, almost parallel and ascending dorsally, extending above anterior surface of spermathecae, then retracing ventrally, curving along inter-posterior surface of spermathecae, finally entering the connecting piece between the spermathecae and bursae. Both primary spermathecae (Sp1) and secondary spermathecae (Sp2) hyaline, surface smooth, with the former located anteromedially to latter. Primary spermathecae globular, moderately large, diameter ca. 1/5–1/4 the width of epigyne; two primary spermathecae close together; spermathecal gland (SG) distinctly small, papilliform, located at the anterolateral surfaces of primary spermathecae. Secondary spermathecae kidney-shaped, anteriorly close together, posteriorly slightly curved and widely separated by ca. 1.6 widths. Fertilisation ducts (FD) membranous, acicular, ca. half primary spermathecae diameter, on their anterior surfaces.

**Male** (*Fig. 2*C and D). Total length 4.43; carapace 1.97 long, 1.48 wide; abdomen 2.46 long, 1.34 wide. Eye sizes and interdistances: AME 0.11, ALE 0.11, PME 0.10, PLE 0.12, AME–AME 0.06, AME–ALE 0.03, PME–PME 0.22, PME–PLE 0.11, MOQL 0.30, MOQA 0.26, MOQP 0.42. Sternum 1.04 long, 0.74 wide. Leg measurements: I 5.81 (1.61, 2.38, 1.23, 0.59), II 6.01 (1.65, 2.47, 1.24, 0.65), III 4.82 (1.36, 1.73, 1.26, 0.47), IV 6.97 (1.97, 2.30, 2.13, 0.57). Leg formula: IV-II-I-III. Cheliceral furrow with six anterior and without posterior teeth.

**Pattern and colouration** (*Fig. 2*C and D). As in females, but body slightly lighter and smaller (see Xin et al. (2020) for others described).

**Figure 3.**
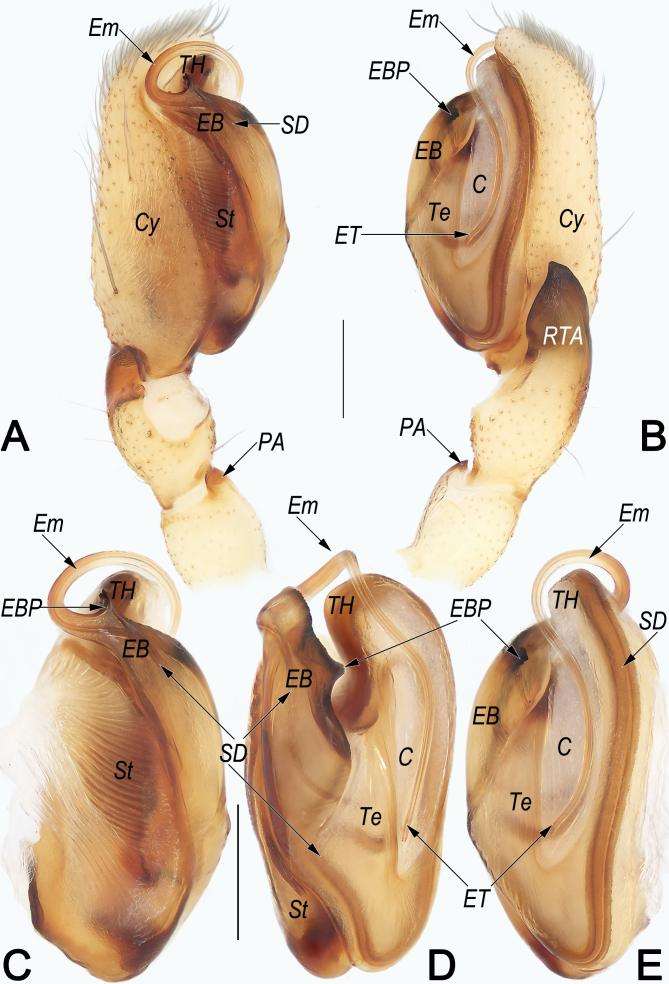
*Clubionahuaban*, male from Guiyang, male palp **A** Prolateral view; **B** Retrolateral view; **C** Bulb, prolateral view; **D** Bulb, ventral view; **E** Bulb, retrolateral view. Abbreviations: C = conductor; Cy = cymbium; EB = embolar base; EBP = embolar base process; Em = embolus; ET = embolar tip; PA = patellar apophysis; RTA = retrolateral tibial apophysis; SD = sperm duct; St = subtegulum; Te = tegulum; TH = tegular hump. Scale bars: 0.2 mm (equal for **A, B**, equal for **C–E**).

**Palp** (Fig. 3A–D). Femur unmodified. Patella with a distinct, thumb-like, small ventral apophysis (PA) originating distally, ca. 1/3 of patella length. Tibia short, ca. 1/3 of cymbium (Cy) length; retrolateral tibial apophysis (RTA) ca. 1.3–1.4× the tibia length, base broad, tip pointed, petal-shaped in retrolateral view. Tegulum (Te) elongated-oval, ca. 2.1 × longer than wide, apically with a distinctly, semicircular hump (TH); sperm duct long and meandering; subtegulum (ST) large, nearly as long as tegulum, prolateral surface partly membranous, wrinkled and ribbed, with numerous diagonal ridges. Embolar base (EB) represented by a long and flat sclerite, inserted at approximately the 8–11 o’clock position of the tegulum, apical margin serrated, medially bearing one subtriangular process (EBP), proximal margin smooth curved; EBP small, its length ca. 1/5 width of embolar base, apex pointing retrolaterally; free part of embolus (Em) slender and flagelliform, bent 90 degrees ventrally and angled across tegular hump, stretched proximally on groove-like conductor, tip (ET) extending basad more than 3/4 length of tegulum, terminating at approximately 4 or 5 o’clock position. Conductor (C) area relatively large, approximately 2/5 tegulum width and 4/5 tegulum length.

**DNAbarcodes**:

5'ATAGTAGGAACGGCTATAAGAGTTTTGATTCGAATGGAATTAGGGCAATCTGGAACATTTTTGGGAGATGATCATCTATATAATGTAGTGGTTACAGCTCATGCTTTTGTAATAATTTTTTTTATAGTGATACCAATTTTGATTGGAGGATTTGGAAATTGAATAGTTCCAATAATATTGGGAGCAGCTGATATAGCTTTTCCTCGAATAAATAATTTGAGATTTTGATTGTTACCTCCTTCTTTATTTTTATTATTTATTTCTTCTATAGCTGAAATGGGAGTTGGAGCAGGATGAACAGTGTATCCTCCTCTTGCTTCTACTGTAGGCCATATAGGGAGAGCTATGGATTTTGCTATTTTTTCTTTACATTTAGCTGGAGCCTCTTCTATTATAGGGGCAGTTAATTTTATTACTACTATTATTAATATACGATCTGTAGGTATGAGTATAGAAAAGGTTCCTTTATTTGTATGATCTGTATTAATTACAGCAGTATTATTATTATTATCTTTACCTGTATTAGCGGGTGCAATTACTATATTATTAACTGATCGTAATTTTAATACTTCTTTCTTTGATCCAGCGGGGGGAGGAGATCCTATTTTATTTCAACATTTATTTTGATTTT3'

(**YHGY403; female from Ya’an; GenBank accession number: PV868257**).

5'ATGGTAGGGACGGCTATAAGAGTTTTGATTCGAATGGAATTAGGTCAATCTGGAACATTTTTGGGAGATGATCATCTATATAATGTAGTGGTTACAGCTCATGCTTTTGTAATAATTTTTTTTATAGTGATACCAATTTTGATTGGAGGATTTGGAAATTGAATAGTTCCAATAATATTGGGAGCAGCTGATATAGCTTTTCCTCGAATGAATAATTTGAGATTTTGATTGTTACCACCTTCTTTATTTTTATTATTTATTTCTTCTATAGCTGAAATGGGAGTTGGAGCAGGATGAACAGTATATCCTCCTCTTGCTTCTACTGTAGGTCATATAGGGAGAGCTATGGATTTTGCTATTTTTTCTTTACATTTAGCTGGAGCCTCTTCTATTATAGGGGCAGTTAATTTTATTACTACTATTATTAATATACGATCTGTAGGTATGAGTATAGAAAAGGTTCCTTTATTTGTATGATCTGTATTAATTACAGCAGTATTATTATTATTATCTTTACCTGTATTAGCAGGTGCAATTACTATATTATTAACTGATCGTAATTTTAATACTTCTTTCTTTGACCCGGCGGGGGGAGGAGATCCTATTTTATTTCAACATTTATTTTGATTTT3'

(**YHGY404; male from Ya’an; GenBank accession number: PV868260**).

5'ATAGTAGGAACGGCTATAAGAGTTTTGATTCGAATGGAATTAGGGCAATCTGGAACATTTTTGGGAGATGATCATCTATATAATGTAGTGGTTACAGCTCATGCTTTTGTAATAATTTTTTTTATAGTAATACCAATTTTGATTGGAGGATTTGGAAATTGAATAGTTCCAATAATATTGGGAGCAGCTGATATAGCTTTTCCTCGAATAAATAATTTGAGATTTTGATTGTTACCTCCTTCTTTATTTTTATTATTTATTTCTTCTATAGCTGAAATGGGAGTTGGAGCAGGATGAACAGTGTATCCTCCTCTTGCTTCTACTGTAGGTCATATAGGGAGAGCTATGGATTTTGCTATTTTTTCTTTACATTTAGCTGGAGCCTCTTCTATTATAGGGGCAGTTAATTTTATTACTACTATTATTAATATACGATCTGTAGGTATGAGTATAGAAAAGGTTCCTTTATTTGTATGATCTGTATTAATTACAGCAGTATTATTATTATTATCTTTACCTGTATTAGCGGGTGCAATTACTATATTATTAACTGATCGTAATTTTAATACTTCTTTCTTTGATCCAGCGGGGGGAGGAGATCCTATTTTATTTCAACATTTATTTTGATTTT3'

(**YHGY405; male from Guiyang; GenBank accession number: PV868258**).

5'ATAGTAGGAACGGCTATAAGAGTTTTGATTCGAATGGAATTAGGGCAATCTGGAACATTTTTGGGAGATGATCATCTATATAATGTAGTGGTTACAGCTCATGCTTTTGTAATAATTTTTTTTATAGTAATACCAATTTTGATTGGAGGATTTGGAAATTGAATAGTTCCAATAATATTGGGAGCAGCTGATATAGCTTTTCCTCGAATAAATAATTTGAGATTTTGATTGTTACCTCCTTCTTTATTTTTATTATTTATTTCTTCTATAGCTGAAATGGGAGTTGGAGCAGGATGAACAGTGTATCCTCCTCTTGCTTCTACTGTAGGTCATATAGGGAGAGCTATGGATTTTGCTATTTTTTCTTTACATTTAGCTGGAGCCTCTTCTATTATAGGGGCAGTTAATTTTATTACTACTATTATTAATATACGATCTGTAGGTATGAGTATAGAAAAGGTTCCTTTATTTGTATGATCTGTATTAATTACAGCAGTATTATTATTATTATCTTTACCTGTATTAGCGGGTGCAATTACTATATTATTAACTGATCGTAATTTTAATACTTCTTTCTTTGATCCAGCGGGGGGAGGAGATCCTATTTTATTTCAACATTTATTTTGATTTT3'

(**YHGY406; female from Guiyang; GenBank accession number: PV868259**).

#### Diagnosis

Females of *C.huaban* are similar to those of *C.baimaensis* Song & Zhu, 1991 by their globular primary spermathecae (Sp1) and copulatory ducts (CD) with similar courses (Fig. 2G and H; Yang et al. (2003): figs. 4A and B; Zhang (2018): figs. 18-1C and D), but can be recognised by: (1) posterior margin of epigynal plate distinctly protruding (vs. slightly protruding) (cf. Fig. 2E and F and Yang et al. (2003): fig. 4A; Zhang (2018): figs. 18-1C); (2) secondary spermathecae (Sp2) kidney-shaped, posteriorly slightly curved (vs. oval, not curved) (cf. Fig. 2G and H and Yang et al. (2003): fig. 4B; Zhang (2018): figs. 18-1D). Males of *C.huaban* also resemble those of *C.baimaensis* Song & Zhu, 1991 in the general shape of the male palp. The palps of both species share similarly-shaped retrolateral tibial apophysis (RTA), but differ in the following: (1) embolar base process (EBP) subtriangular, inserted at retrolateral rim of embolar base (EB) (vs. tooth-shaped, inserted at anterior rim of EB) (cf. Fig. 3B, D and E and Yang et al. (2003): fig. 4D; Zhang (2018) figs. 18-1E); (2) embolus (E) relatively longer, extending basally for more than 3/4 tegulum length and terminating at approximately 4 or 5 o’clock position (vs. shorter, extending basally for ca. 1/2 tegulum length and terminating at approximately 3 o’clock position) (cf. Fig. 3B, D and E and Yang et al. (2003): fig. 4D; Zhang (2018) figs. 18-1E and G); (3) conductor (C) area relatively longer, approximately 4/5 tegulum length (vs. shorter, approximately 2/3 tegulum length) (cf. Fig. 3B, D and E and Yang et al. (2003): fig. 4D; Zhang (2018) figs. 18-1E and G).

#### Distribution

China (Guizhou, Sichuan). The new collections extend the known range of this species by ~ 510 km to the northwest (Yucheng District of Ya’an City, Sichuan) from the type locality (Xiangzhigou Scenic Spot of Guiyang City, Guizhou) (Fig. 1).

## Analysis

**Figure 4.**
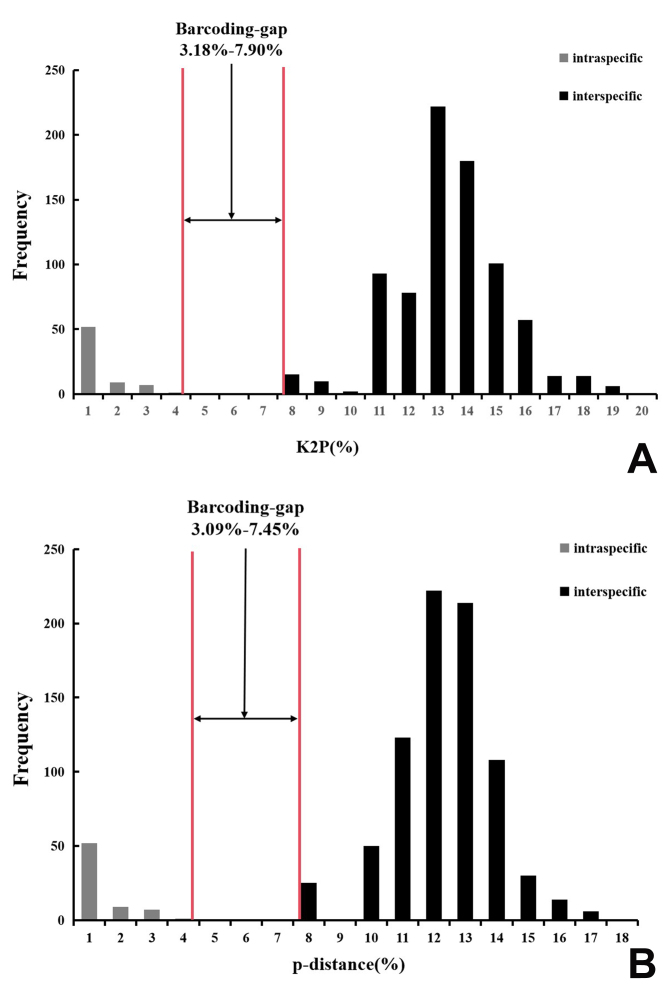
DNA barcoding gap for the *Clubionatrivialis* group. Histograms show the division of intraspecific (grey) and interspecific (black) CO1 sequence variation, based on the Kimura two-parameter (K2P) (A) and uncorrected p-distance (B).

When assigning 12 species of *trivialis*-group, a distinct gap between intraspecific and interspecific genetic distances was found, ranging from 3.18% to 7.90% for K2P (Fig. 4A; Suppl. material 2) and from 3.10% to 7.45% for p-distance (Fig. 4B; Suppl. material 2). The lowest mean interspecific distance was 8.02%/7.56% (K2P/uncorrected p-distance) found between *C.pygmaea* and *C.quebecana* and the highest mean intraspecific distance was 3.18%/3.09% (K2P/uncorrected p-distance) was estimated for *C.moesta* (Suppl. material 3). As a result, a total of 42 sequences were identified as belonging to 12 species by the barcoding gap range, consistent with the morphological results. Amongst them, four newly-sequenced samples presumed to be *C.huaban* were identified as conspecific. Our results indicate that even single-locus analyses, based on the COI barcodes, when integrated with morphological data and collection experience, may provide sufficiently reliable species delimitation for the *Clubionatrivialis* group.

## Supplementary Material

XML Treatment for Clubiona huaban

610BB991-C565-584F-A3DE-C30E07A5A21D 10.3897/BDJ.13.e157384.suppl1 Supplementary material 1 A list of current *Clubionatrivialis*-group species in alphabetical order Data type List Brief description A list of current *Clubionatrivialis*-group species, with GenBank accession numbers of samples used in species molecular delimitation in this study. File: oo_1366659.xls https://binary.pensoft.net/file/1366659 Hao Yu

36CE5230-62B0-52B6-9904-A28772778C74 10.3897/BDJ.13.e157384.suppl2 Supplementary material 2 Estimates of Evolutionary Divergence between Sequences Data type MatrixOutput Brief description Estimates of Evolutionary Divergence between Sequences using Kimura 2-parameter and p-distance models. File: oo_1356364.xls https://binary.pensoft.net/file/1356364 Hao Yu

D7FFAF4B-5860-50DB-B63E-A9F54237EDA6 10.3897/BDJ.13.e157384.suppl3 Supplementary material 3 Intraspecific and interspecific genetic distances Data type Table Brief description Intraspecific and interspecific genetic distances in CO1 for 12 Clubionatrivialis-group species. File: oo_1356365.xlsx https://binary.pensoft.net/file/1356365 Hao Yu

## Figures and Tables

**Table 1.** Voucher samples of *C.huaban*: specimen label, sex, sample collection locality with coordinates and GenBank accession numbers.

Specimen code	Sex	Locality	Coordinates	Elevation (m a.s.l.)	COI GenBank accession
YHCLU0403	♀	Zhougongshan National Forest Park, Yucheng District, Ya’an City, Sichuan Pro. (new distribution record)	29.96°N, 103.05°E	650	PV868257
YHCLU0404	♂	Zhougongshan National Forest Park, Yucheng District, Ya’an City, Sichuan Pro. (new distribution record)	29.96°N, 103.05°E	650	PV868260
YHCLU0405	♂	Xiangzhigou Scenic Spot, Wudang District, Guiyang City, Guizhou Pro. (type locality)	26.79°N, 106.92°E	1115	PV868258
YHCLU0406	♀	Xiangzhigou Scenic Spot, Wudang District, Guiyang City, Guizhou Pro. (type locality)	26.79°N, 106.92°E	1115	PV868259
